# Supplementary material for: Identification of photoperiod-induced specific miRNAs in the adrenal glands of Sunite sheep (Ovis aries)
Source: Front Vet Sci. 2022 Jul 22;9:888207. doi: 10.3389/fvets.2022.888207 (PMC9354845; doi:10.3389/fvets.2022.888207)
Supplement: Supplementary file 1 [file Data_Sheet_1.ZIP › Supplementary file/miRDeep2 file.zip!/miRDeep2 file/Know_Structure/SP42A2/oar-mir-200a.pdf]

miRBase precursor	: oar-mir-200a
Total read count	: 267
oar-miR-200a read count	: 260
remaining reads	: 7

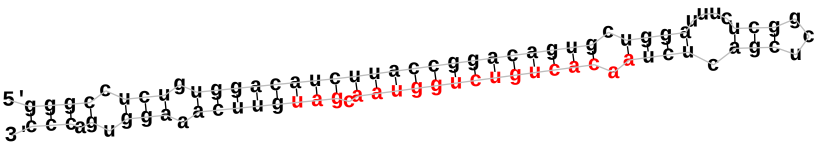

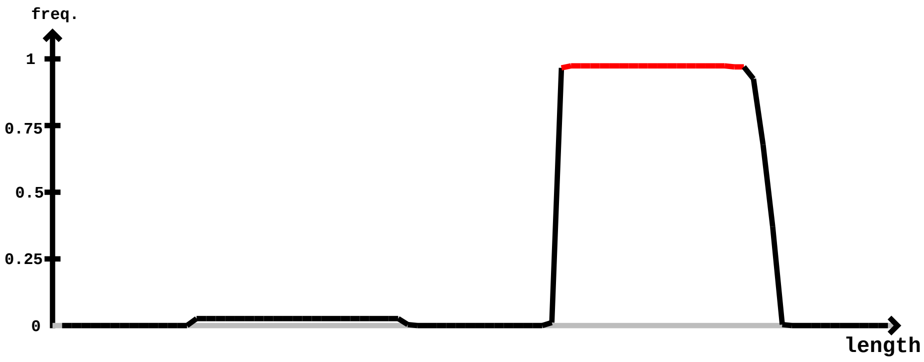

oar-miR-200a

```
5'- gggcucuguggacaucuuaccggacagugcuggauuucucggcucgacucuaacacugucugguaacgauguucaaaggugaccc  -3'    exp
    ((((.(((.((((((((((((((((((((.((((....(((...))).)))).))))))))))))).)))))))).))).).)))    reads      mm         sample
    ............caucuuaccggacagugcugga...................................................    6          0          seq
    ............caucuuaccggacagugcuggau..................................................    1          0          seq
    .................................................cuaacacugucugguaaAgau...............    1          1          seq
    .................................................cuaacacugucugguaaAgaug..............    1          1          seq
    .................................................cuaacacugucugguaacgaug..............    1          0          seq
    ..................................................uaacacugucugguaacg.................    1          0          seq
    ..................................................uaacacugucugguaaAgau...............    3          1          seq
    ..................................................uaacacugucugguaacgau...............    8          0          seq
    ..................................................uaacacugucugguaacgaug..............    44         0          seq
    ..................................................uaacacugucugguaaAgaug..............    20         1          seq
    ..................................................uaacacugucugguaacgaugu.............    82         0          seq
    ..................................................uaacacugucugguaacgauguu............    96         0          seq
    ..................................................uaacacugucugguaacgauguuc...........    1          0          seq
    ...................................................aacacugucugguaacgauguu............    2          0          seq
```
